# Inoculation and co-inoculation of lettuce and arugula hydroponically influence nitrogen metabolism, plant growth, nutrient acquisition and photosynthesis

**DOI:** 10.3389/fpls.2025.1547821

**Published:** 2025-04-16

**Authors:** Carlos Eduardo da Silva Oliveira, Thaissa Julyanne Soares Sena Oliveira, Arshad Jalal, Guilherme Carlos Fernandes, Andréa de Castro Bastos, Marcelo Rinaldi da Silva, Gabriela Rodrigues Sant’Ana, Jailson Vieira Aguilar, Liliane Santos de Camargos, Tiago Zoz, Marcelo Carvalho Minhoto Teixeira Filho

**Affiliations:** ^1^ Department of Agronomy, State University of Mato Grosso do Sul – UEMS, Cassilândia, Mato Grosso do Sul, Brazil; ^2^ Department of Plant Protection, Rural Engineering and Soils, São Paulo State University (UNESP), School of Engineering, Ilha Solteira, São Paulo, Brazil; ^3^ The BioActives Lab, Center for Desert Agriculture (CDA) Division of Biological and Environmental Sciences (BESE) King Abdullah University of Science and Technology (KAUST), Thuwal, Saudi Arabia; ^4^ Salesian Auxilium Catholic University Center, Araçatuba, São Paulo, Brazil; ^5^ Department of Biology and Zootechny, São Paulo State University (UNESP), School of Engineering, Ilha Solteira, São Paulo, Brazil; ^6^ Department of Agronomy, State University of Mato Grosso do Sul – UEMS, Mundo Novol, Mato Grosso do Sul, Brazil

**Keywords:** amino acid content, carbohydrate content, carbon assimilation, chlorophyll, nitrate reductase, plant growth-promoting bacteria, photosynthetic efficiency

## Abstract

The objective of this study was to investigate the effects of single and combined inoculations of *A. brasilense, B. subtilis* and *P. fluorescens* on lettuce and arugula grown in a hydroponic system. The study was carried out in a greenhouse and was designed in randomized blocks with five replications. The treatments consisted of inoculation with *A. brasilense, B. subtilis* and *P. fluorescens* and co-inoculation with *A. brasilense + B. subtilis, A. brasilense + P. fluorescens, B. subtilis + P. fluorescens* and *A. brasilense + B. subtilis + P. fluorescens* via nutrient solution. An increase in the length and fresh mass of the shoot and leaf chlorophyll concentrations of arugula and lettuce was observed under co-inoculations of *A. brasilense + P. fluorescens* and *B. subtilis + P. fluorescens*. Greater length, fresh mass and volume of the lettuce root system were observed under the co-inoculations of *A. brasilense + B. subtilis, A. brasilense + P. fluorescens* and *B. subtilis + P. fluorescens* in arugula under the inoculations of *A. brasilense* and *A. brasilense + P. fluorescens.* Greater nitrate reductase activity was detected in leaves, and lower nitrate accumulation was detected in lettuce and arugula under inoculations with *A. brasilense, P. fluorescens* and *B. subtilis + P. fluorescens*. The greatest accumulation of N, P, K, Ca and Mg in the lettuce shoot was obtained under inoculation with *P. fluorescens*, *A. brasilense + P. fluorescens* and *B. subtilis + P. fluorescens*. Co-inoculation with *A. brasilense + P. fluorescens* and *B. subtilis + P. fluorescens* was the most efficient combination for increasing the growth, nutrient acquisition and functioning of nitrogen metabolism in arugula lettuce plants.

## 1 Introduction

Lettuce (*Lactuca sativa* L.) is a low-sodium salad vegetable, low-fat, low-calorie, abundant source of health-promoting compounds (vitamins, flavonoids, glycosylated phytochemicals, sesquiterpene lactones (lactucopic and lactucin), hydroxycinnomic acids, carotenoids, vitamins-B, vitamin-C, tocopherol and a rich source of iron (Fe). In addition, secondary metabolites in lettuce leaves may be associated with many beneficial health factors, including substances that scavenge free radicals, cardiovascular disease, inflammation, cysts, and diabetes (Yang et al., 2022). Arugula (*Eruca sativa* Mill.) is widely consumed as a fresh salad because of its high contents of vitamin C, Fe, and calcium (Ca). Additionally, it offers detoxifying properties beneficial to human health, attributed to its antioxidant compounds, including antigenotoxic agents, polyphenols, and glucosinolates (Tassi et al., 2018). Arugula and lettuce are among the most popular and widely used green vegetables and are grown extensively in hydroponic systems (Oliveira et al., 2023a, 2024). In 2022, lettuce sold worldwide had revenue of US$3.44 billion, which represents 0.015% of all global food trade, and arugula sold worldwide had revenue of US$211.2 million, with leafy vegetables having greater global economic representation (OEC, 2024).

Vegetable growers are increasingly adjusting to new technology without sacrificing financial returns because the demand for premium products is growing sustainably. Plants grown in hydroponic systems are becoming more climatically tolerant owing to the nutrient film approach (Oliveira et al., 2023b). According to Dalastra et al. (2020), it also enhances the control of pests and diseases, increases productivity and precocity, and makes plants, particularly vegetables, more tolerant of varying climates. However, nitrogen in the nitric form is the most commonly used form of nitrogen in hydroponic cultivation systems because of its low volatility and toxicity for absorption by plants, in addition to its toxic effect on plants in which high concentrations of nitrogen in the ammoniacal form are inserted into the nutrient solution (Zhu et al., 2021). The increased absorption of nutrients by arugula and lettuce plants is desirable for achieving high yields of leaves with greater efficiency, and increased nitrogen accumulation in the shoots of plants is associated with increased carbon assimilation; photosynthetic activity; and concentrations of carbohydrates, chlorophyll and amino acids (Baslam et al., 2020; Peinado-Torrubia et al., 2023).

Increased assimilation of N-NO_3_^-^ by plants and its transport to leaf tissues can improve growth, photosynthetic activity and carbon assimilation via photosynthesis for the structural and metabolic functions of nitrogen in plants (Cunha et al., 2024). However, owing to the nitrate metabolic route within the plant, high energy expenditure is needed to synthesize nitric-N into ammonium-N, resulting in amino acids, chlorophyll, proteins and nucleic acids (Oliveira et al., 2024). Furthermore, the increase in leaf nitrate accumulation is a negative indicator of food organoleptic quality because of the increase in the bitterness of foods and the risks to human health caused by excessive nitrate intake (Gato et al., 2023; Oliveira et al., 2024). Given these difficulties in changing the source of N supplied to plants in a hydroponic system, it is necessary to adopt measures that can reduce the amount of nitrate in plant tissues without harming growth, photosynthetic metabolism or the absorption of other nutrients.

Inoculation with strains of bacteria from the *Azospirillum* genus increases growth promotion through greater production of plant hormones, root growth, and photosynthetic activity and increased N–NO_3_^-^ synthesis through greater activity of the enzyme nitrate reductase (Gato et al., 2023; Oliveira et al., 2023b; Vendruscolo et al., 2024), such as when inoculated with strains of *Pseudomonas* that promote biological control of diseases, increased absorption of nutrients, synthesis of amino acids and the activity of the antioxidant system and nitrate reduction, such as increased production of plant hormones (Oliveira et al., 2023a, 2024; Putra et al., 2024), such as when inoculated with *Bacillus* strains that promote increased photosynthetic activity, carbon assimilation, nutrient absorption, synthesis of amino acids, proteins and the activity of nitrate reductase enzymes (Oliveira et al., 2023c; Wang et al., 2023; Plocek et al., 2024).

In other studies, it was possible to highlight the use of microorganisms such as *Trichoderma harzianum, Azospirillum brasilense, Pseudomonas fluorescens, P. migulae, P. lundensis, Bacillus subtilis, B. amyloliquefaciens* and *Arthrobacter pascens* as isolated inoculants and rarely together in the same solution in the cultivation of different vegetables in a hydroponic system, making it possible to verify increased plant growth, nutrient accumulation, photosynthetic activity, and activity of enzymes involved in nitrogen metabolism and hormone production under such inoculations (Moreira et al., 2022; Gato et al., 2023. Oliveira et al., 2023a, b, c; Wang et al., 2023; Oliveira et al., 2024; Plocek et al., 2024; Putra et al., 2024). In this sense, it is important to verify the interaction of these microorganisms in isolated and combined forms via nutrient solution and to define whether a synergistic or antagonistic effect occurs between the microorganisms, considering the different functions and actions of these species in plants. The objective of this research was to investigate the effects of the inoculation and co-inoculation of *A. brasilense, P. fluorescens* and *B. subtilis* via nutrient solution on growth, nutrient accumulation, gas exchange and leaf nitrate reductase activity of lettuce and arugula in a hydroponic system.

## 2 Materials and methods

### 2.1 Experimental characterization

The research was carried out via a hydroponic lettuce and arugula cultivation system employing the nutrient film technique (NFT) method within a greenhouse covered with 30% shade cloth. This study was conducted at the School of Engineering, São Paulo State University, Ilha Solteira, Brazil. The precise location was at coordinates 20°25’07” S, 51°20’31” W, with an altitude of 376 m. Meteorological information was collected from the UNESP automatic meteorological station during the period spanning March 30^th^ to April 30^th^, 2022 (**Supplementary Figure 1**).

### 2.2 Experimental design

The experiment was designed in randomized blocks with five replications. Eight plants of each species were collected per plot for the analysis. The eight treatments consisted of inoculation via nutrient solution (1. non-inoculated, inoculations with 2. *Pseudomonas fluorescens*, 3. *Bacillus subtilis*, 4. *Azospirillum brasilense*, and co-inoculations with 5. A*. brasilense + P. fluorescens, 6. A. brasilense + B. subtilis*, 7. *B. subtilis + P. fluorescens* and 8. A*. brasilense + B. subtilis + P. fluorescens*) via nutrient solution at the time of implantation. The inoculants used were *Azospirillum brasilense* strains Ab-V5 and Ab-V6 (2x10^8^ unit forming colonies (UFC) mL^-1^) at a dose of 0.44 mL L^-1^ of inoculant (Oliveira et al., 2023b), *Bacillus subtilis* strain CCTB04 (1×10^8^ UFC mL^-1^) at a dose of 0.32 mL L^-1^ of inoculant (Oliveira et al., 2023c) and *Pseudomonas fluorescens* strain CCTB03 (2×10^8^ UFC mL^-1^) at a dose of 0.29 mL L^-1^ of inoculant (Oliveira et al., 2024). This isolation was sent to the UNESP microbiology laboratory for multiplication according to methodology Bettiol et al. (2005).

### 2.3 Implementation and conduct of the experiment

The description of the NFT hydroponic benches, the lettuce cultivar ‘Angelina’, arugula cultivar ‘Astro’, the production of seedlings, The nutrient solution composed of concentrated fertilizers from Hidrogood Fert was used at a concentration of (0.666 g L^−1^) indicated for all stages of crop development, with the following nutrient concentrations: 10% N, 9% phosphorus (P), 28% potassium (K), 4.3% sulfur (S), 3.3% magnesium (Mg), 0.06% boron (B), 0.01% copper (Cu), 0.05% manganese (Mn), 0.07% molybdenum (Mo), and 0.02% zinc (Zn). Also, calcium nitrate (15.5% N and 26.5% calcium) at 0.495 g L^−1^ and Hidrogood Fert Iron EDDHA (6% iron) at 0.020 g L^−1^ were used, to achieve electrical conductivity (EC) of 1.5 dS m^-1^. EC and pH were measured and adjusted daily in the morning. On this occasion, the EC was readjusted to the EC determined for each reservoir of nutrient solution with the replacement of fertilizers, if necessary, and to maintain the pH between 6.0 and 6.5, sulfuric acid (25%) was used when pH was above 6.5, and sodium hydroxide (25%) when pH was below 6.0, was described by Oliveira et al. (2023a) and Oliveira et al. (2023b).

### 2.4 Biometric assessments

The assessments were conducted for 31 days post-transplantation of lettuce and arugula seedlings, which marked the harvest period. Each plot contained twenty-four plants, and the eight central plants were collected, and the fresh matter of both the root system and shoot of each lettuce and arugula plant was individually examined via a precision scale with an accuracy of 0.001 kg. The numbers of leaves of lettuce and arugula were counted manually. The collected samples were dried in an air-forced oven at 60°C for 72 hours to determine the dry matter of the roots and shoots, utilizing an analytical scale with a precision of 0.001 g. The lengths of both the shoots and the roots were measured in centimeters using a millimeter ruler with a precision of 1 mm. The root volume was measured by a volumetric cylinder on the basis of the displacement of the volume of internal water in the cylinder.

### 2.5 Nutritional assessments

The plant materials were ground in a Wiley mill, and the N, P, K, Ca, Mg and S concentrations in the shoots and roots of lettuce and arugula were determined at harvest (31 days after transplanting) following the methodology outlined by Malavolta et al. (1997). The concentrations of NO_3_^-^ and NH_4_^+^ in the shoots and roots were assessed using the procedure described by Silva (2009). The accumulation of nutrients in the shoots and roots of the plants was calculated on the basis of the respective dry matter and nutrient concentrations of N, P, K, Ca, Mg, S, NO_3_^-^, and NH_4_^+^ from the current experiment, employing the following Equation 1:


(1) $$NA\;g\;m^{-2}\;mg\;m^{-2}=DM(kg\;m^{-2})*NC\;g\;kg^{-1}or\;mg\;kg^{-1}\;$$

NA: nutrient accumulation;

DM: dry matter;

NC: nutrient concentration.

### 2.6 Physiological assessments

*In vivo* nitrate reductase (*NR*) activity was measured according to the method of Radin (1974). It third fully unfolded leaf was collected for enzymatic and leaf chlorophyll analysis in the morning (9–10 am) to avoid light intensity interference. Fresh leaf tissue samples were collected, stored on ice in plastic bags, and transported to the laboratory. The material was washed with deionized water. One hundred milligrams of freshly sliced tissue was transferred to a test tube containing 3 ml of phosphate buffer (50 mmol L^-1^ + 200 mmol L^-1^ KNO_3_) at pH 7.4.

To improve the penetration of the solution into the tissue, these samples were vacuum infiltrated for two minutes. The tubes were then shielded from light by being wrapped in aluminum foil and incubated in a 33°C water bath for 30 min. The reaction was stopped by adding 1 mL of 1% sulfanilamide in 2 L^-1^ HCl solution followed by 1 mL of 0.05% naphthylenediamine solution. The produced nitrite (NO_2_^-^) was measured spectrophotometrically (UV-5100, Metash, Shanghai, China) at 540 nm via a standard nitrite calibration curve. The enzyme activity was directly related to the amount of NO_2,_ and the results were expressed in μmol NO_2_^-^ g^-1^ h^-1^ fresh weight (FW).

The chlorophyll a (Chl a), b (Chl b), total (Chl T) and carotenoid (CAR) contents were determined using the methodology by Hiscox and Israelstam (1979), as described in Aguilar et al. (2023). After the samples were measured with a spectrophotometer (UV-5100, Metash, Shanghai, China), the contents of the photosynthetic pigments were calculated and expressed in mg g^-1^ FW via the following equations:


Chl a=(12.70×ABS663)-(2.69×ABS645)

Chl b=(22.90×ABS645)−(4.68×ABS663)

Chl T=(20.20×ABS645)+(8.02×ABS663)

CAR=((1000×ABS470)−(1.29×Chl a−53.38×Chl b)÷198)

Total amino acids were quantified with ninhydrin acid using the methodology by Yemm et al. (1955) as described in Aguilar et al. (2023). The determination was carried out in a spectrophotometer (UV-5100, Metash, Shanghai, China) at λ = 570 nm, and the results are expressed in μmol g^-1^ FW. The total carbohydrate content was determined according to the methodology by Yemm and Willis (1954) as described in Aguilar et al. (2023). The concentration of total carbohydrates was measured using standard curve of glucose solution, and the results are expressed in µmol g^-1^ FW.

The assessment of gas exchange was conducted on eight plants per plot during the harvest period, specifically between 9 a.m. and 11 a.m. The evaluation focused on fully expanded leaves situated in the central part of each plant, using an infrared gas analyzer (IRGA, CIRAS-3, PP Systems, Amesbury, MA, USA). The intercellular CO_2_ concentration (*Ci* - μmol CO_2_ mol^-1^ air^-1^), stomatal conductance (*gs* - mmol of H_2_O m^-2^ s^-1^), transpiration (*E* - mmol of H_2_O m^-2^ s^-1^), net photosynthesis rate (*A* - μmol CO_2_ m^-2^ s^-1^), and water use efficiency (*WUE* - mmol CO_2_ mol^-1^ H_2_O^-1^) were evaluated.

### 2.7 Statistical analysis

All variables demonstrated a normal distribution and homogeneous variances, as confirmed by the Shapiro–Wilk test (Shapiro and Wilk, 1965). The data were subjected to analysis of variance, and the significance of the obtained mean squares was assessed using the F-test at a 5% probability level. The inoculations and co-inoculations were compared using the Scott–Knott test at the 5% probability level to evaluate the means using SigmaPlot 16 software (Systat, 2025).

## 3 Results

### 3.1 Lettuce

#### 3.1.1 Effects of inoculations and co-inoculations on root growth

There was a significant (p ≤ 0.01) effect of inoculation with plant growth-promoting bacteria on the root length, root volume, and root fresh and dry mass of the lettuce plants in the hydroponic system (**Supplementary Table 1**).

**Figure 1:**
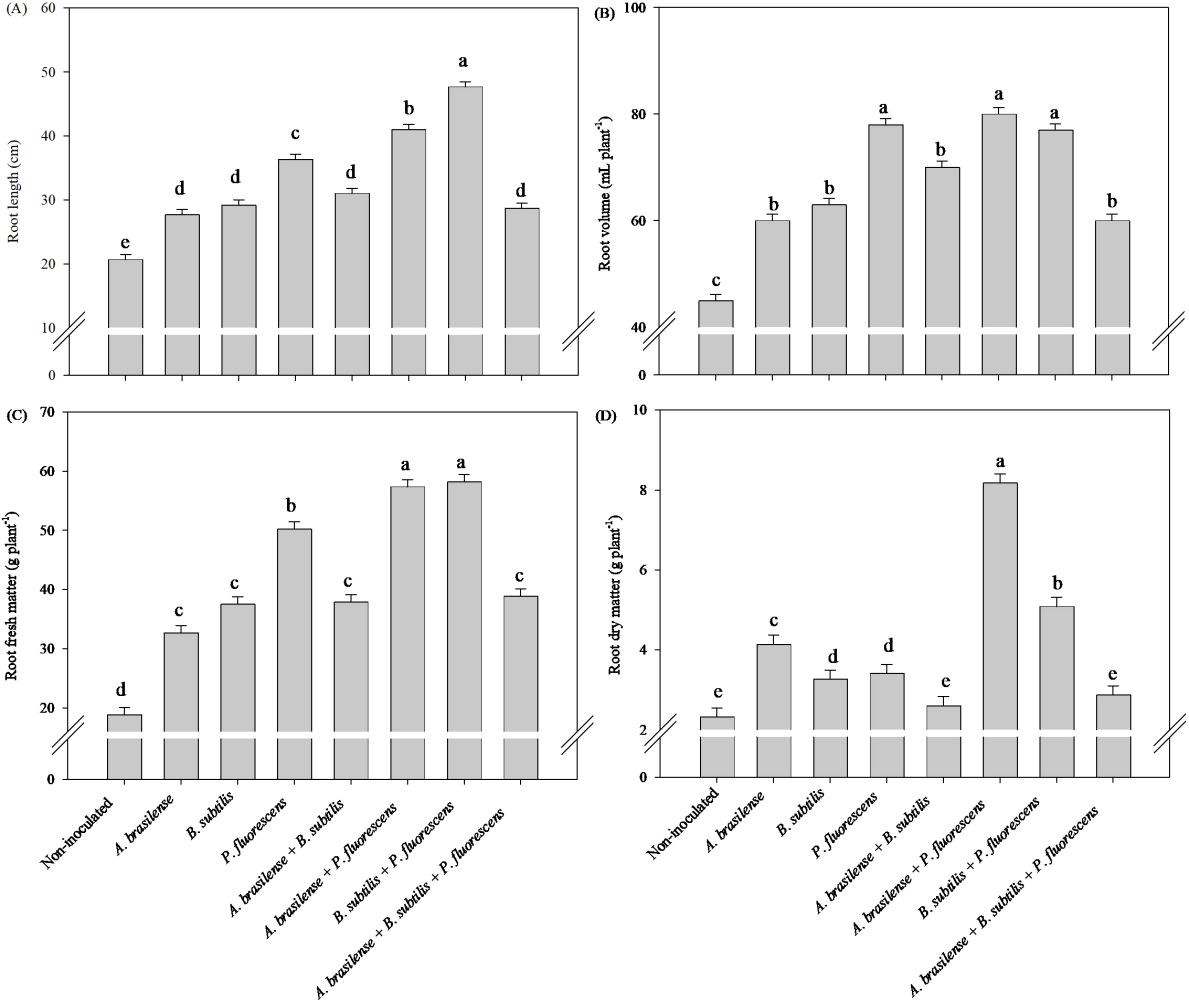
Effect of inoculations with growth-promoting bacteria on root length **(A)**, root volume **(B)**, root fresh matter **(C)** and root dry matter **(D)** of hydroponic lettuce plants.

The greatest root length was observed under the co-inoculation of *B. subtilis + P. fluorescens* with an increase of 130% in relation to the non-inoculated treatment (**Figure 1A**). The inoculation of *P. fluorescens* and the co-inoculations of *A. brasilense + P. fluorescens* and *B. subtilis + P. fluorescens* increased the root volume in 73, 77 and 71% of lettuce plants compared to the non-inoculated, respectively (**Figure 1B**). The inoculation of *P. fluorescens* and the co-inoculations of *A. brasilense + P. fluorescens* and *B. subtilis + P. fluorescens* an increased the root fresh mass by 166, 204 and 208% of lettuce plants compared to the non-inoculated, respectively (**Figure 1C**), and the highest root dry mass was obtained under the co-inoculation of *A. brasilense + P. fluorescens* with an increased in 260% compared with the other inoculations (**Figure 1D**).

#### 3.1.2 Effects of inoculations and co-inoculations on shoot growth

There was a significant (p ≤ 0.01) effect of inoculations on the shoot length, number of leaves, shoot fresh mass and dry mass of the shoots of the lettuce plants in the hydroponic system (**Supplementary Table 2**).

**Figure 2:**
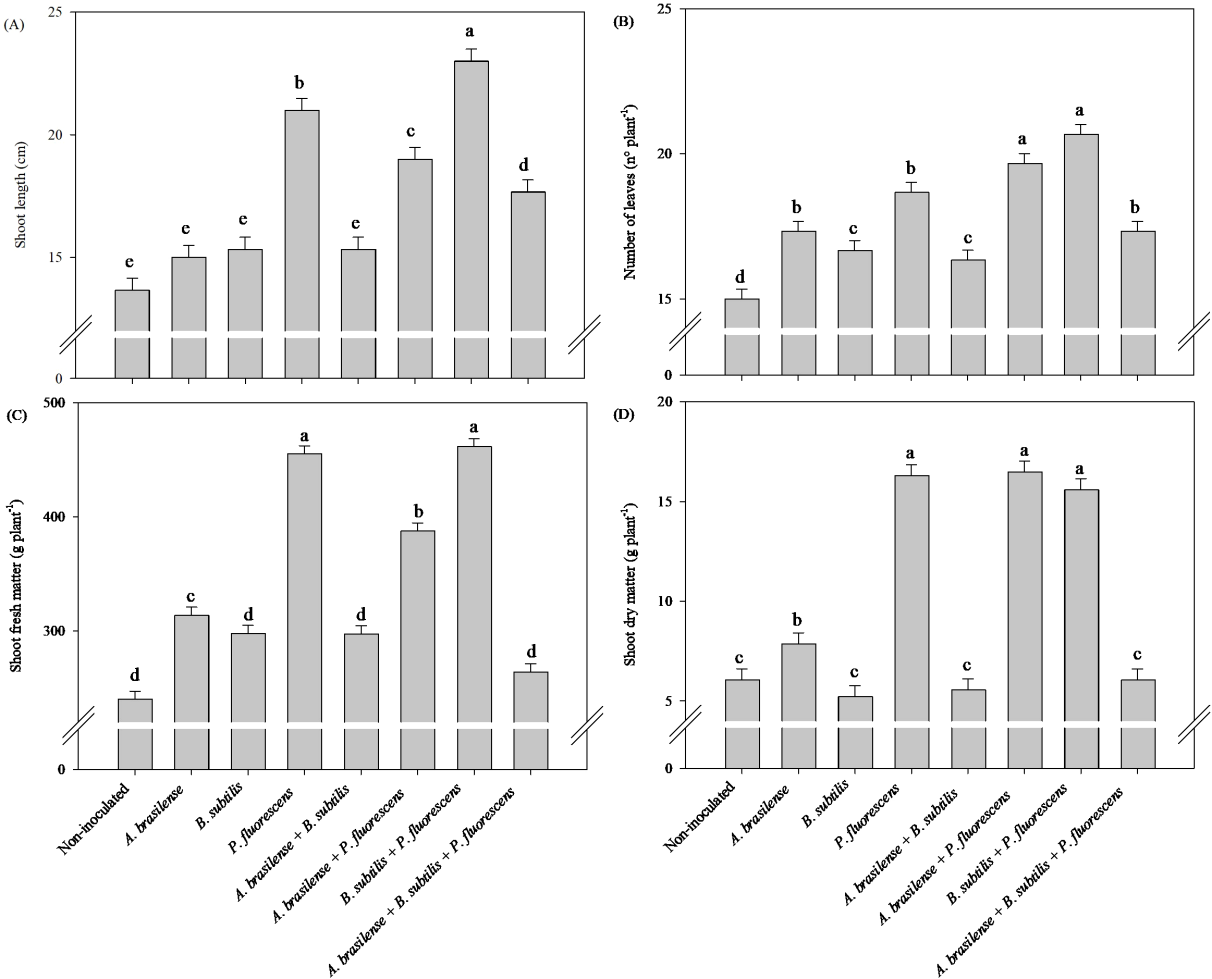
Effects of inoculations with growth-promoting bacteria on shoot length **(A)**, number of leaves **(B)**, shoot fresh matter **(C)** and shoot dry matter **(D)** of hydroponic lettuce plants.

Greater shoot length were observed under co-inoculation with *B. subtilis + P. fluorescens* with an increased in 68% in relation to non-inoculated lettuce plants, respectively (**Figure 2A**). However, the highest shoot fresh mass was observed under inoculation with *P. fluorescens* and co-inoculation with *B. subtilis + P. fluorescens* with an increase in 89% and 92% in relation to non-inoculated lettuce plants, respectively (**Figure 2C**). Co-inoculation with *A. brasilense + P. fluorescens* and *B. subtilis + P. fluorescens* resulted an increase in 31% and 37% in number of leaves of lettuce plant compared to the non-inoculated, respectively (**Figure 2B**). Greater shoot dry matter was detected in the lettuce plants inoculated with *P. fluorescens* and co-inoculated with *A. brasilense + P. fluorescens* and *B. subtilis + P. fluorescens* with an increase in 161%, 163% and 160% compared non-inoculated plants, respectively (**Figure 2D**).

#### 3.1.3 Effects of inoculations and co-inoculations on nutrient accumulation

There was a significant (p ≤ 0.01) effect of inoculation on the accumulation of N, P, K, Ca, Mg and S in the shoots of the lettuce plants (**Supplementary Table 3**).

**Figure 3:**
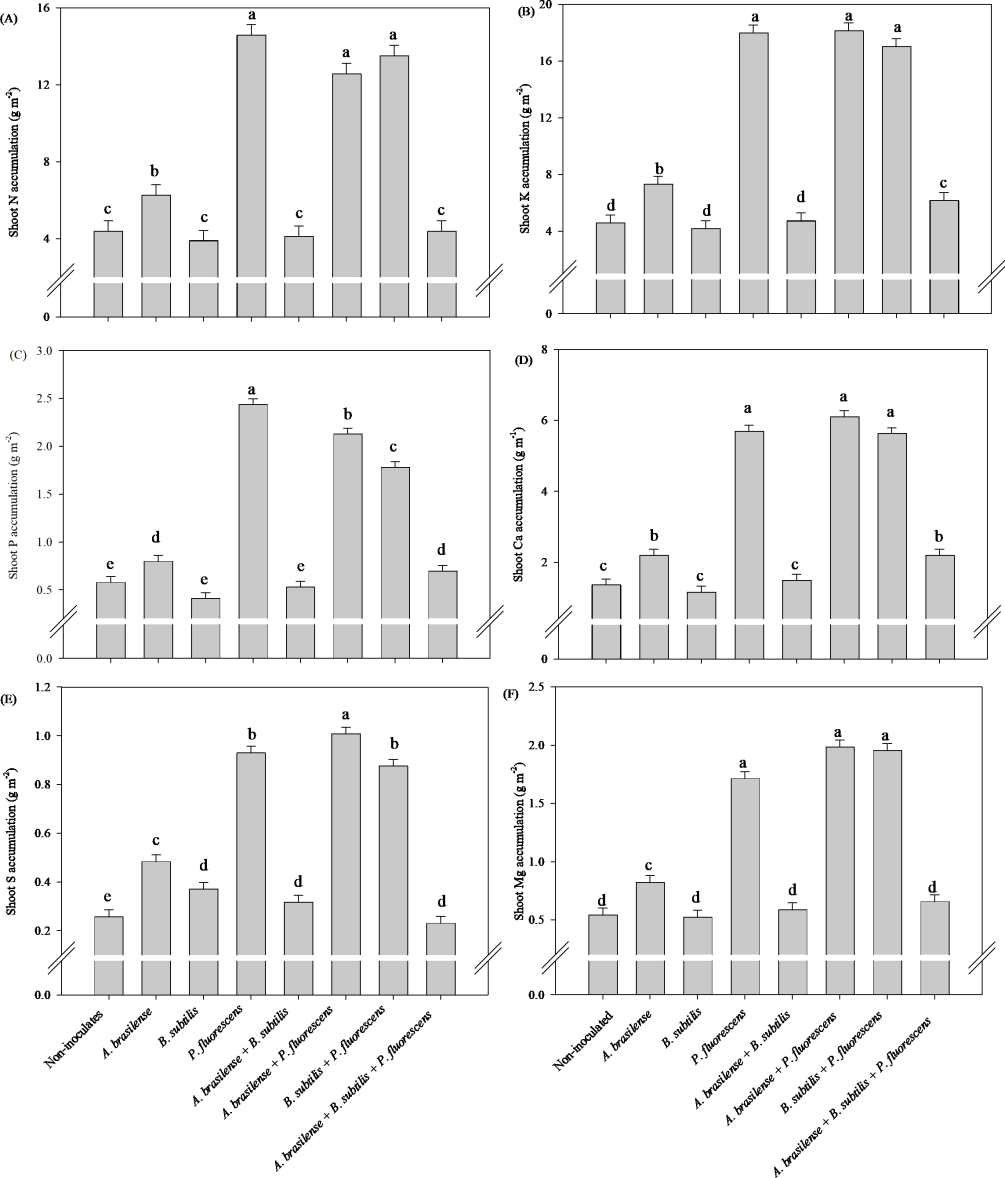
Effects of inoculations with growth-promoting bacteria on shoot nitrogen **(A)** potassium **(B)**, phosphorus **(C)**, calcium **(D)**, sulfur **(E)** and magnesium **(F)** accumulation in lettuce plants.

The inoculation with *P. fluorescens* and co-inoculation with *A. brasilense + P. fluorescens* and *B. subtilis + P. fluorescens* provided an increase in 233%, 187% and 208% in shoot N accumulation; 293%, 296% and 272% increases in shoot K accumulation; 319%, 350% and 314% in shoot Ca accumulation; and 216%, 266% and 260% in shoot Mg accumulation in lettuce under inoculations with *P. fluorescens*, *A. brasilense + P. fluorescens* and *B. subtilis + P. fluorescens* in comparison to non-inoculated, respectively (**Figures 3A, B, D, F**). Greater shoot P accumulation of lettuce plants was observed with inoculation of *P. fluorescens* with and increase in 322% compared to non-inoculated plants (**Figure 3C**). However, the co-inoculation with *A. brasilense + P. fluorescens* provided increase in 233% in shoot S accumulation in relation to non-inoculated lettuce plants (**Figure 3E**).

#### 3.1.4 Effects of inoculations and co-inoculations on nitrogen metabolism

There was a significant (p ≤ 0.01) effect of inoculations on the accumulation of N-NH_4_^+^ and N-NO_3_^-^ in shoots and roots, leaf NR activity, leaf amino acids (TAA) and leaf carbohydrate (TC) concentrations of lettuce plants in a hydroponic system (**Supplementary Table 4**).

**Figure 4:**
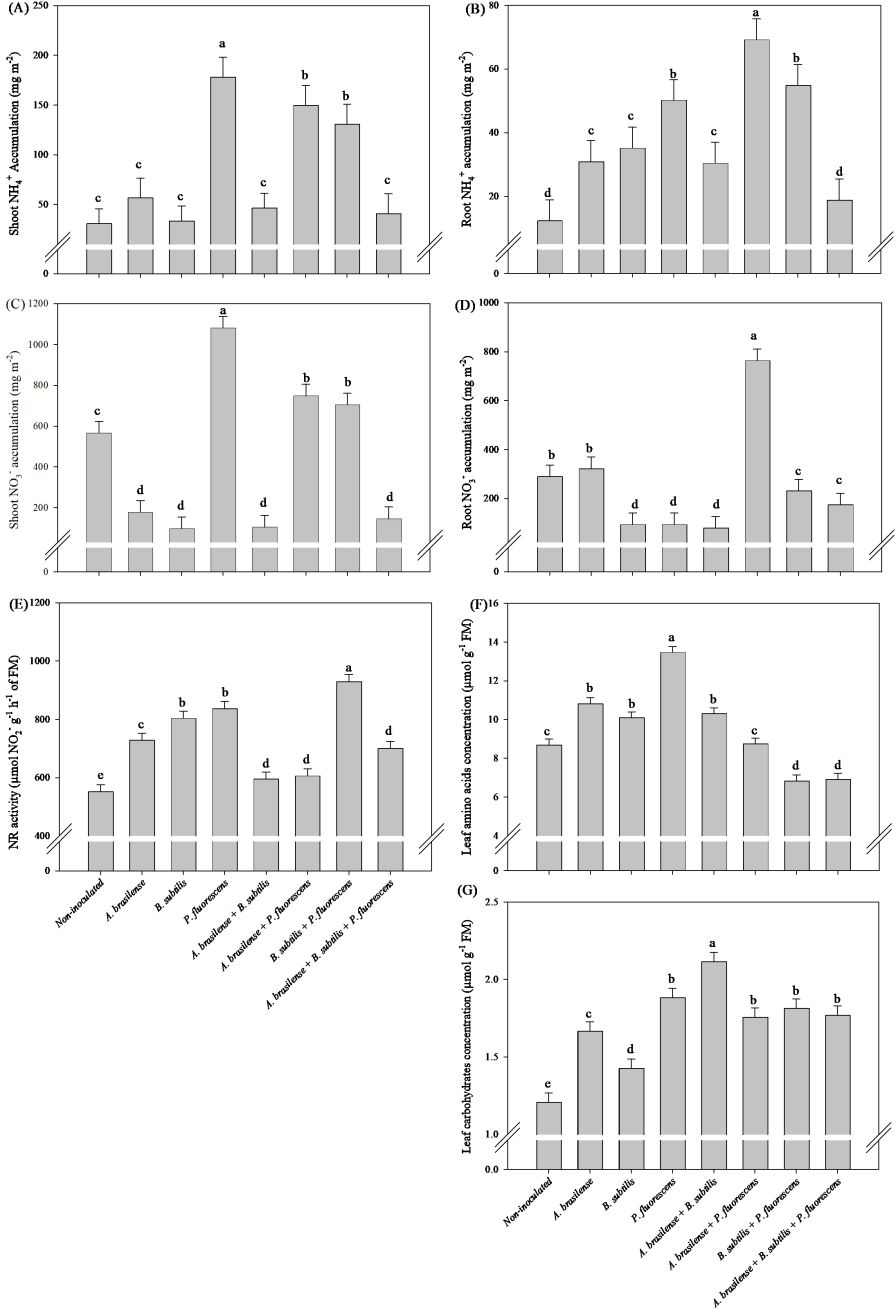
Effects of inoculations with growth-promoting bacteria on ammonium (NH_4_^+^) accumulation in shoot **(A)** and root **(B)**, nitrate (NO_3_^-^) accumulation in shoot **(C)** and root **(D)**, nitrate reductase (NR) activity **(E)**, leaf amino acids concentration **(F)** and leaf carbohydrate concentration **(G)** in lettuce plants.

Inoculation with *P. fluorescens* increase in 55% the TAA, in 479% in shoot NH_4_^+^ accumulation in relation non-inoculated lettuce plants, however, the decreases of 219%, 479%, 437% and 287% shoot NO_3_^-^ accumulation under inoculation with *A. brasilense* and co-inoculations with *A. brasilense + B. subtilis* and *A. brasilense + B. subtilis + P. fluorescens* compared non-inoculated lettuce plants, respectively (**Figures 4A, C, F**). Co-inoculation with *A. brasilense + P. fluorescens* promoted greater accumulation of NH_4_^+^ and NO_3_^-^ in root in relation to other treatments (**Figures 4B, D**). Greater leaf NR activity was detected in the lettuce plants co-inoculated with *B. subtilis + P. fluorescens* with an increase in 68% in relation non-inoculated plants; however, all the inoculated and co-inoculated plants presented greater NR activity in the leaves than the non-inoculated plants (**Figure 4E**). Co-inoculation with *A. brasilense + B. subtilis* promoted increase in 53% the TC leaves content in relation to non-inoculated lettuce plants (**Figure 4G**).

#### 3.1.5 Effects of inoculations and co-inoculations on leaf chlorophyll and gas exchange

There was a significant (p ≤ 0.01) effect of inoculations on the chlorophyll A (Chl a), chlorophyll B (Chl b), chlorophyll total (Chl T) and carotenoids (CAR) concentrations in the leaves of the lettuce plants in the hydroponic system (**Supplementary Table 5**).

**Figure 5:**
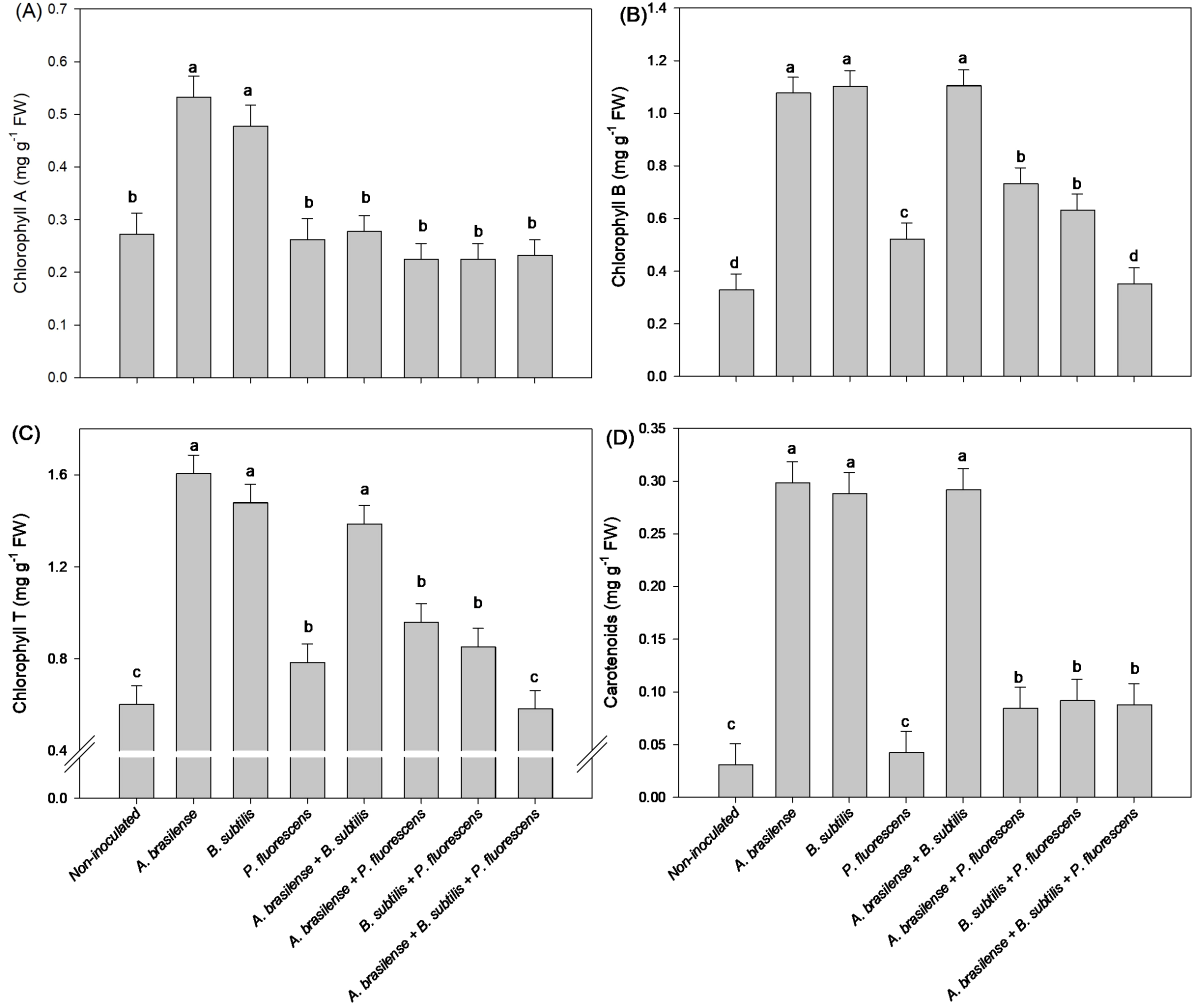
Effects of inoculations with growth-promoting bacteria on chlorophyll A **(A)**, chlorophyll B **(B)**, chlorophyll total (T) **(C)** and carotenoids **(D)** concentration in leaves in lettuce plants.

The isolated inoculation of *A. brasilense* and *B. subtilis* provided an increase in 100 and 88% in concentration of Chl a in lettuce leaves compared to the non-inoculated plants, respectively (**Figure 5A**). However, the isolated inoculation of *A. brasilense* and *B. subtilis* as well as the co-inoculation of *A. brasilense + B. subtilis* provided an increase in 265, 234 and 235% in Chl b; 166, 146 and 130% in Chl T; and 860, 826 and 840% in the CAR concentration in lettuce leaves compared to non-inoculated plants, respectively (**Figures 5B–D**).

There was a significant (p ≤ 0.01) effect of inoculations on the intercellular CO_2_ concentration (*Ci*), net photosynthesis rate (*A*), stomatal conductance (*gs*), transpiration (*E*) and water use efficiency (*WUE*) in the leaves of the lettuce plants in the hydroponic system (**Supplementary Table 6**).

**Figure 6:**
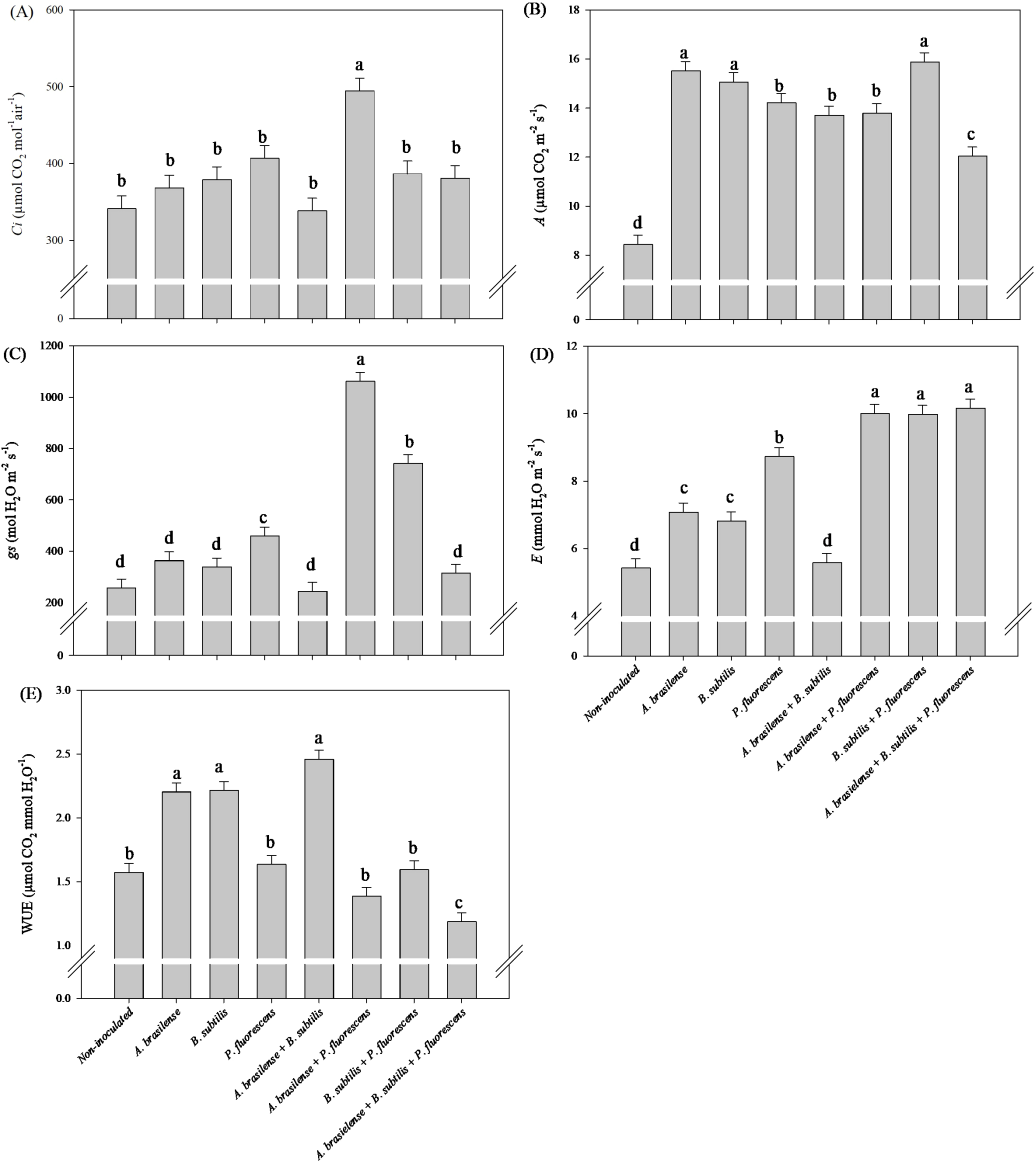
Effects of inoculations with growth-promoting bacteria on intercellular CO_2_ concentration (*Ci*) **(A)**, net photosynthesis rate (*A*) **(B)**, stomatal conductance (*gs*) **(C)**, transpiration (*E*) **(D)** and water use efficiency (*WUE*) **(E)** in leaves of lettuce plants.

The highest *Ci* and *gs* in lettuce leaves was observed under the co-inoculation of A*. brasilense + P. fluorescens* with an increase in45% and 314% compared to the non-inoculated plants, respectively (**Figures 6A, C**). However, inoculation with *A. brasilense* and *B. subtilis* and co-inoculation with *B. subtilis + P. fluorescens* provided an increase in 84%, 78% and 88% in *A* in the leaves of lettuce compared non-inoculated, respectively (**Figure 6B**). The co-inoculations with *A. brasilense + B. subtilis + P. fluorescens*, *A. brasilense + P. fluorescens* and *B. subtilis + P. fluorescens* resulted an increase in 70%, 70% and 71% in *E* in the leaves of the lettuce plants in comparison to the non-inoculated, respectively (**Figure 6D**). However, inoculation with *A. brasilense, B. subtilis* and co-inoculation with *A. brasilense + B. subtilis* provided an increase in 40%, 41% and 56% in *WUE* in the leaves of lettuce in relation non-inoculated plants, respectively (**Figure 6E**).

### 3.2 Arugula

#### 3.2.1 Effects of inoculations and co-inoculations on root growth

There was a significant (p ≤ 0.01) effect of inoculations with plant growth-promoting bacteria on root length, root volume, root fresh and dry mass of arugula plants in hydroponic system (**Supplementary Table 1**).

**Figure 7:**
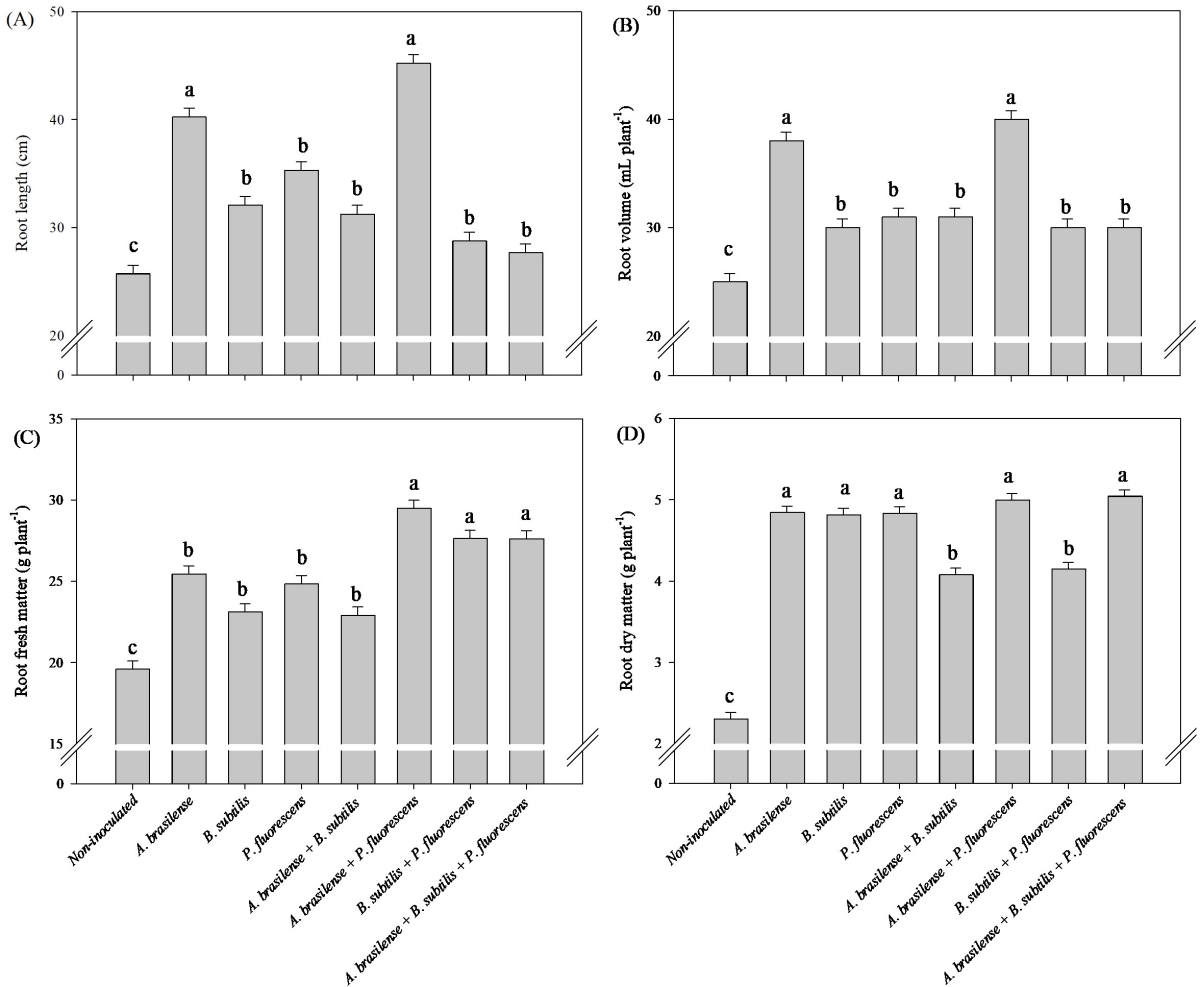
Effect of inoculations with growth-promoting bacteria on root length **(A)**, root volume **(B)**, root fresh matter **(C)** and root dry matter **(D)** of hydroponic arugula plants.

Compared with the other treatments, the inoculation of *A. brasilense* and the co-inoculation of *A. brasilense + P. fluorescens* provided an increase of 57% in root length and 75% in root volume of arugula plants in relation to the non-inoculated plants, respectively (**Figures 7A, B**). Co-inoculations of *A. brasilense + P. fluorescens*, *B. subtilis + P. fluorescens* and *A. brasilense + B. subtilis + P. fluorescens* resulted an increase of 50%, 40% and 41% in root fresh mass of arugula compared to the non-inoculated, respectively (**Figure 7C**). However, inoculations of *A. brasilense, B. subtilis, P. fluorescens* and co-inoculations of *A. brasilense + P. fluorescens* and *A. brasilense + B. subtilis + P. fluorescens* provided an increase in 109%, 108%, 108%, 117% and 117% in root dry mass of arugula in comparison to the non-inoculated, respectively (**Figure 7D**).

#### 3.2.2 Effects of inoculations and co-inoculations on shoot growth

There was a significant (p ≤ 0.01) effect of inoculations on the shoot length, number of leaves, shoot fresh mass and dry mass of the shoots of arugula plants in the hydroponic system (**Supplementary Table 2**).

**Figure 8:**
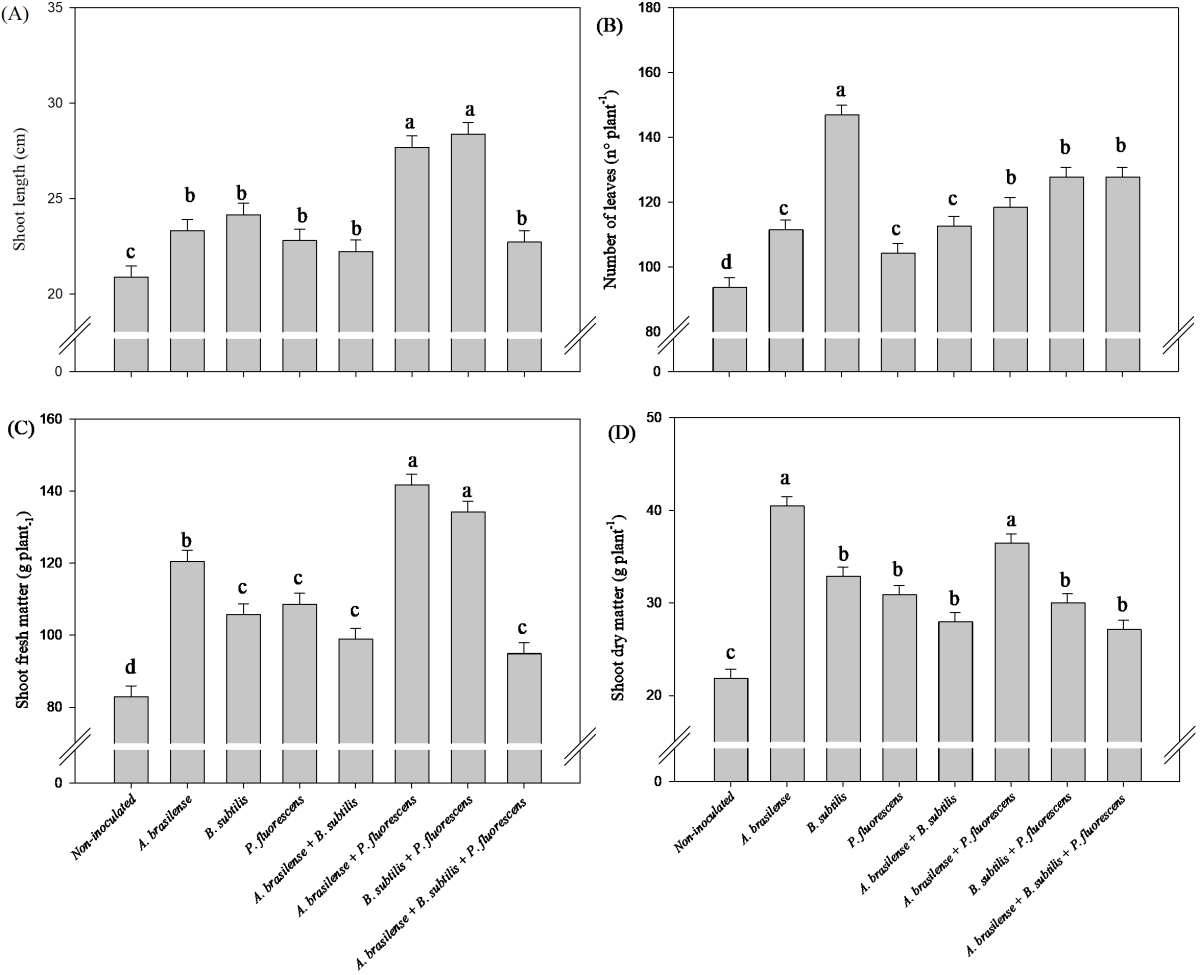
Effects of inoculations with growth-promoting bacteria on shoot length **(A)**, number of leaves **(B)**, shoot fresh matter **(C)** and shoot dry matter **(D)** of hydroponic arugula plants.

The greatest shoot length and shoot fresh mass of the arugula plants were obtained under the co-inoculations of *A. brasilense + P. fluorescens* and *B. subtilis + P. fluorescens* with an increase of 32% and 36% in shoot length and 71% and 62% in shoot fresh mass in relation to the non-inoculated plants, respectively (**Figures 8A, C**). Inoculation with *B. subtilis* provided an increase of 56% in number of leaves on arugula plants compared to non-inoculated plants (**Figure 8B**). Inoculation with *A. brasilense* and co-inoculation with *A. brasilense + P. fluorescens* resulted and increase of 85% and 67% in shoot dry mass of arugula plants in relation to the non-inoculated plants, respectively (**Figure 8D**).

#### 3.2.3 Effects of inoculations and co-inoculations on nutrient accumulation

There was a significant (p ≤ 0.01) effect of inoculations on the accumulation of N, P, K, Ca, Mg and S in the shoots of arugula plants (**Supplementary Table 3**).

**Figure 9:**
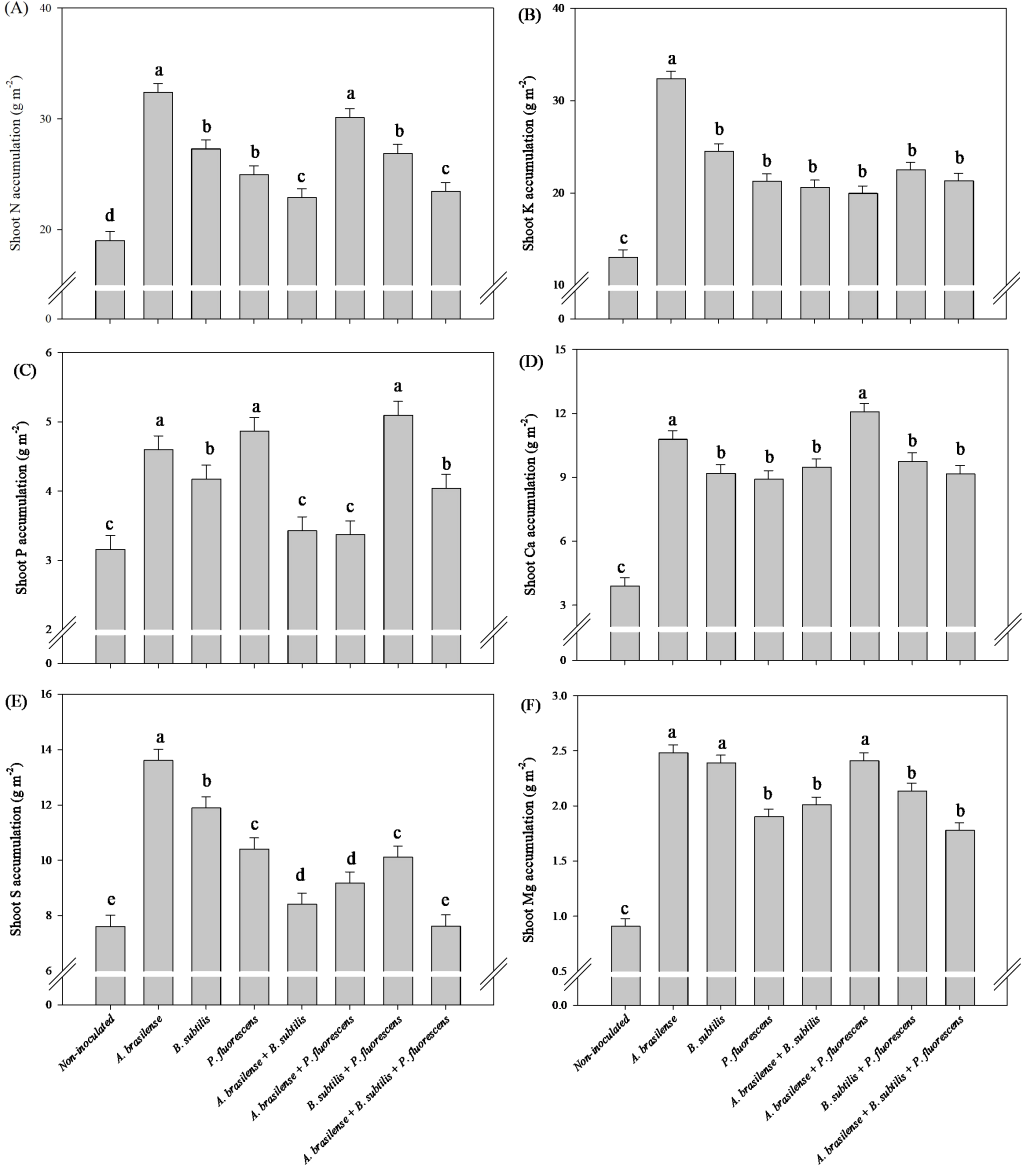
Effects of inoculations with growth-promoting bacteria on shoot nitrogen **(A)** potassium **(B)**, phosphorus **(C)**, calcium **(D)**, sulfur **(E)** and magnesium **(F)** accumulation in arugula plants.

The greatest accumulation of shoot N and Ca accumulation in arugula plants was observed under inoculation with *A. brasilense* and co-inoculation with *A. brasilense + P. fluorescens* with an increase of 70% and 58% in N accumulation; 176% and 209% in Ca accumulation compared to the non-inoculated, respectively (**Figures 9A, D**). However, inoculation with *A. brasilense* resulted an increase of 148% in shoot K accumulation and 79% in shoot S accumulation of arugula plants in relation to the non-inoculated, respectively (**Figures 9B, E**). Inoculation with *A. brasilense*, *P. fluorescens* and co-inoculation with *B. subtilis + P. fluorescens* provided an increase of 45%, 54% and 61% in shoot P accumulation of arugula plants compared to non-inoculated, respectively (**Figure 9C**). Inoculation with *A. brasilense*, *B. subtilis* and co-inoculation with *A. brasilense + P. fluorescens* an increase of 173%, 163% and 165% in shoot Mg accumulation of arugula plants compared to non-inoculated, respectively (**Figure 9F**).

#### 3.2.4 Effects of inoculations and co-inoculations on nitrogen metabolism

There was a significant (p ≤ 0.01) effect of inoculations on the accumulation of N-NH_4_^+^ and N-NO_3_^-^ in shoots and roots, leaf NR activity, leaf TAA and TC concentration of arugula plants in a hydroponic system (**Supplementary Table 4**).

**Figure 10:**
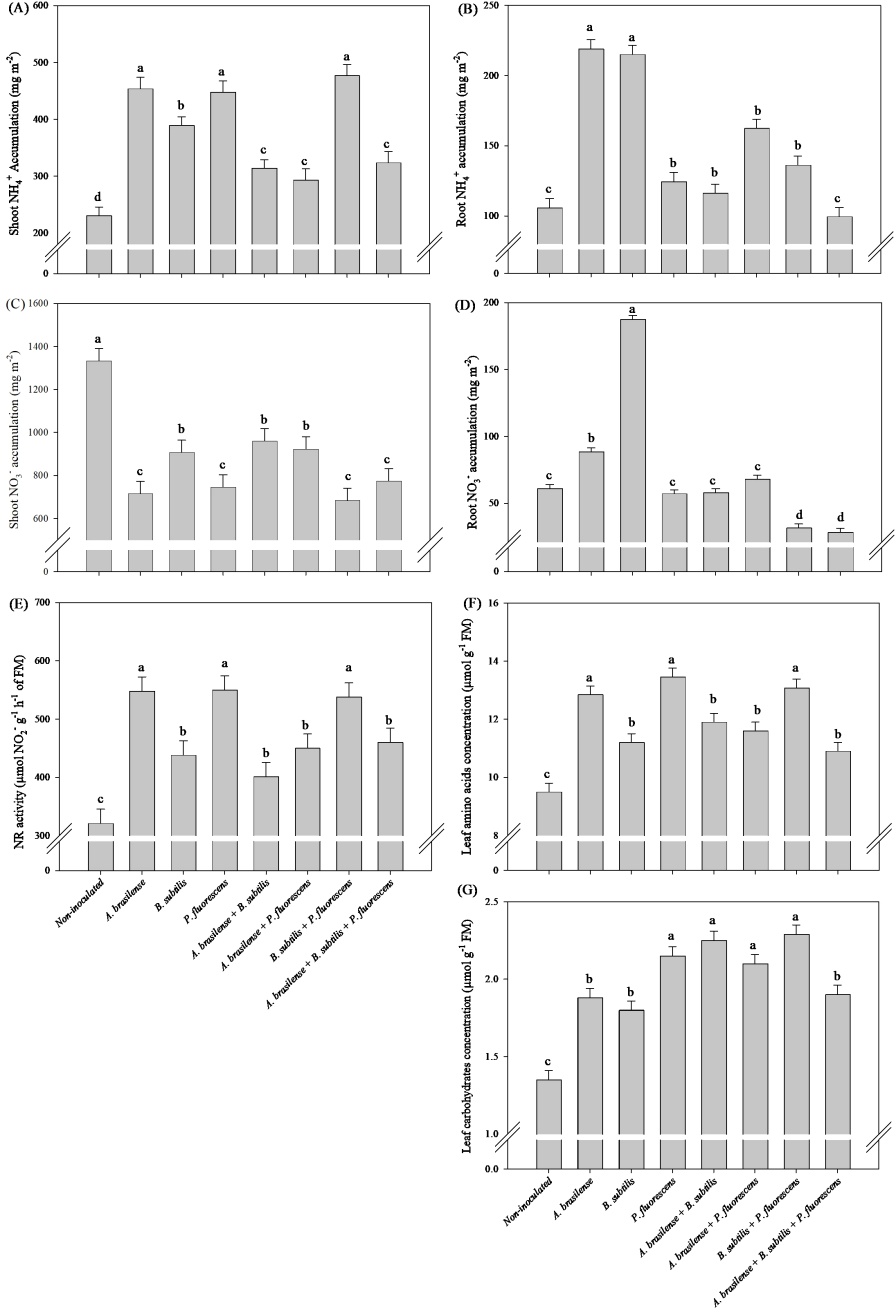
Effects of inoculations with growth-promoting bacteria on ammonium (NH_4_^+^) accumulation in shoot **(A)** and root **(B)**, nitrate (NO_3_^-^) accumulation in shoot **(C)** and root **(D)**, nitrate reductase (NR) activity **(E)**, leaf amino acids concentration **(F)** and leaf carbohydrate concentration **(G)** in arugula plants.

The inoculation with *A. brasilense, P. fluorescens* and the co-inoculation of *B. subtilis + P. fluorescens* provided an increase of 90%, 94% and 107% in shoot NH_4_^+^ accumulation, 71%, 71% and 68% in leaf NR activity and 35%, 42% and 38% in leaf TAA concentration of arugula plants in comparison to the non-inoculated plants, respectively (**Figures 10A, E, F**). The greatest amount of shoot NO_3_^-^ accumulation in arugula plants was observed under the inoculation of non-inoculation in comparison to the inoculations, however, a decreased of 86, 79 and 95% in shoot NO_3_^-^ accumulation was observed under the inoculation of *A. brasilense, P. fluorescens* and the co-inoculation of *B. subtilis + P. fluorescens* compared to the non-inoculated, respectively (**Figure 10C**). All inoculation and co-inoculation led to an increase in the leaf TC concentration in arugula plants in relation to the non-inoculated treatment, however, the inoculation with *P. fluorescens* and co-inoculations with *A. brasilense + B. subtilis, A. brasilense + P. fluorescens* and *B. subtilis + P. fluorescens* provided an increase of 59%, 66%, 55% and 69% in TC concentration in leaves of arugula in comparison non-inoculated plants, respectively (**Figure 10G**). Inoculation with *A. brasilense* and *B. subtilis* provided greater root NH_4_^+^ accumulation in arugula compared to other inoculations (**Figure 10B**). Inoculation with *B. subtilis* resulted in greater root NO_3_^-^ accumulation in arugula compared to other inoculations (**Figure 10D**).

#### 3.2.5 Effects of inoculations and co-inoculations on leaf chlorophyll and gas exchange

There was a significant (p ≤ 0.01) effect of inoculations on the Chl a, Chl b, Chl T and CAR concentrations in the leaves of arugula plants in the hydroponic system (**Supplementary Table 5**).

**Figure 11:**
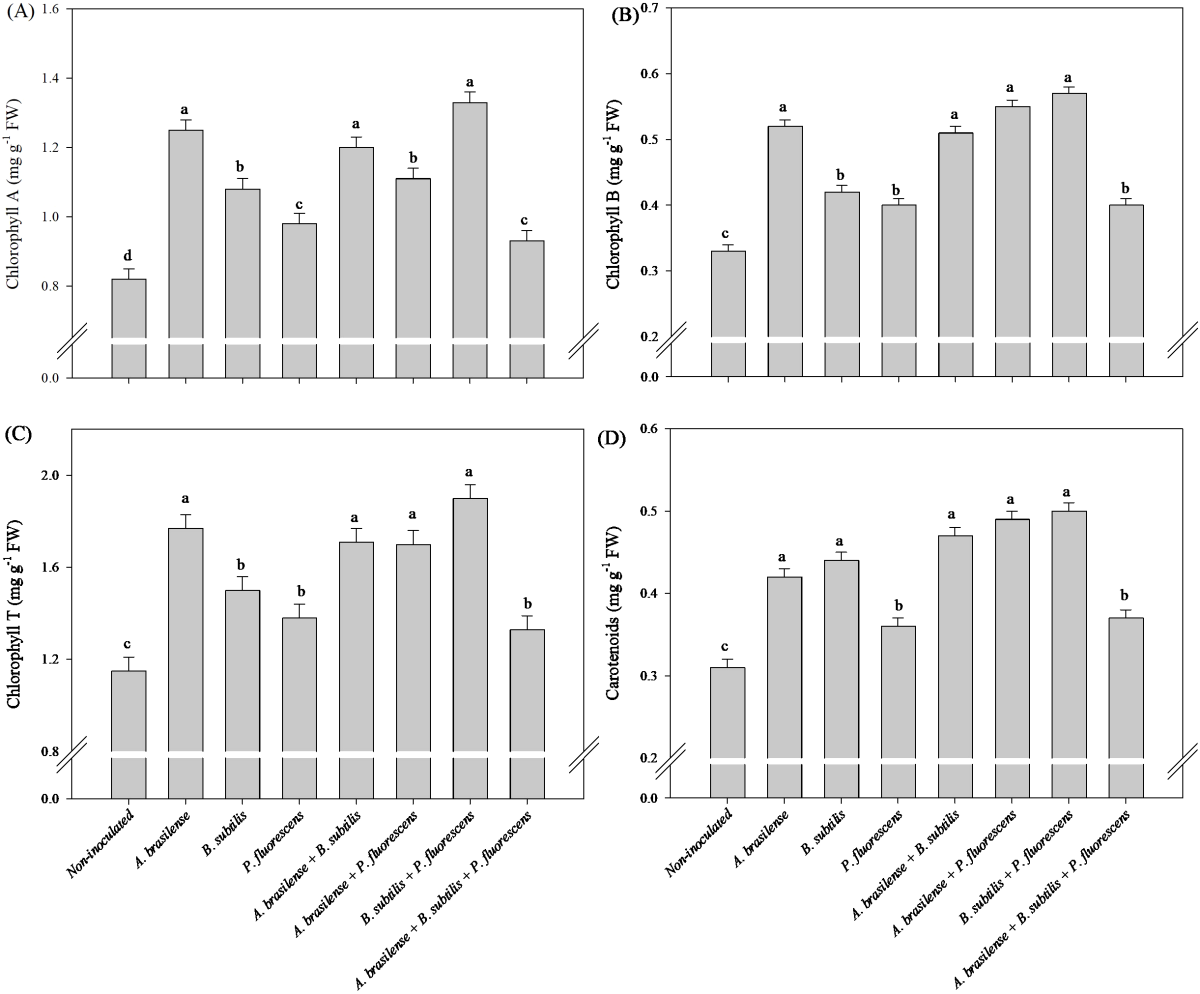
Effects of inoculations with growth-promoting bacteria on chlorophyll A **(A)**, chlorophyll B **(B)**, chlorophyll total (T) **(C)** and carotenoids **(D)** concentration in leaves in arugula plants.

The inoculation with *A. brasilense* and co-inoculation with *A. brasilense + B. subtilis* and *B. subtilis + P. fluroescens* resulted an increase in 52%, 46%, and 62% in concentrations of Chl a in the leaves of the arugula plants in comparison to non-inoculated, respectively (**Figure 11A**). The highest leaf concentration of Chl b and Chl T of arugula were detected under inoculation with *A. brasilense* and the co-inoculations of *A. brasilense + B. subtilis, A. brasilense + P. fluorescens* and *B. subtilis + P. fluorescens* with an increase in 57%, 54%, 66% and 72% in Chl b; and 54%, 49%, 48% and 65% in the Chl T in comparison to non-inoculated, respectively (**Figures 11B, C**). However, the highest leaf concentration of CAR of arugula was observed under the inoculation of *A. brasilense, B. subtilis* and the co-inoculations of *A. brasilense + B. subtilis, A. brasilense + P. fluorescens* and *B. subtilis + P. fluroescens* with an increase in 35%, 41%, 51%, 58% and 61% in comparison to the non-inoculated plants, respectively (**Figure 11D**).

There was a significant (p ≤ 0.01) effect of inoculations on the *Ci*, *A*, *gs*, *E* and *WUE* in the leaves of arugula plants in the hydroponic system (**Supplementary Table 6**).

**Figure 12:**
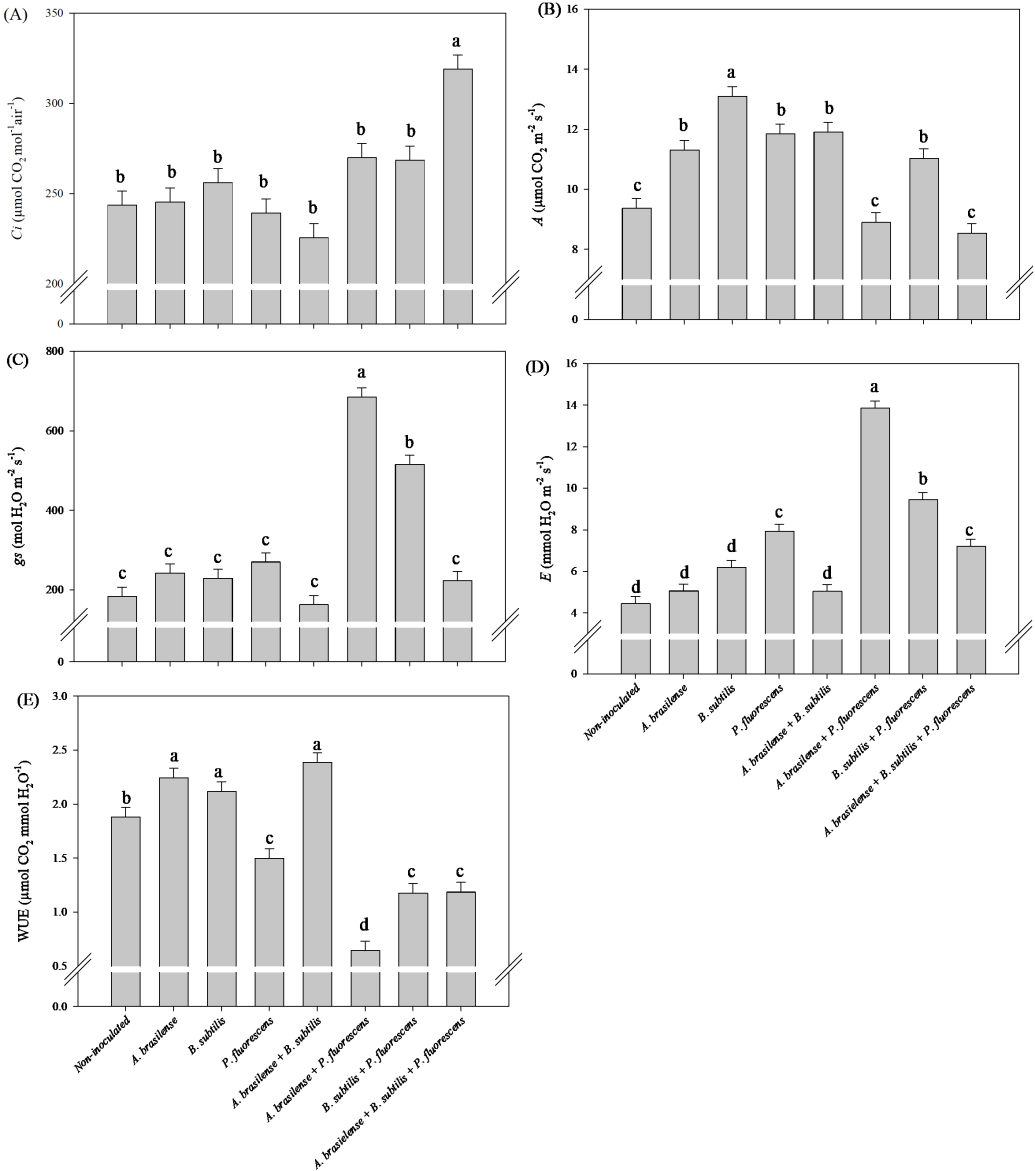
Effects of inoculations with growth-promoting bacteria on intercellular CO_2_ concentration (*Ci*) **(A)**, net photosynthesis rate (*A*) **(B)**, stomatal conductance (*gs*) **(C)**, transpiration (*E*) **(D)** and water use efficiency (*WUE*) **(E)** in leaves of arugula plants.

The highest *Ci* in arugula leaves was observed under the co-inoculation of *A. brasilense + B. subtilis + P. fluorescens* with an increase in 31% compared to non-inoculated (**Figure 12A**). However, inoculation with *B. subtilis* led to an increase in 18% in *A* in arugula leaves in relation to the non-inoculated plants (**Figure 12B**). Furthermore, the co-inoculation with *A. brasilense + P. fluorescens* resulted an increase in 274% in *gs* and 212% in *E* in the leaves of arugula compared to the non-inoculated plants (**Figures 12C, D**). Inoculation with *A. brasilense, B. subtilis* and co-inoculation with *A. brasilense +B. subtilis* improved an increase in 19%, 13% and 27% in *WUE* in the leaves of arugula plants in comparison with non-inoculated plants, respectively (**Figure 12E**).

## 4 Discussion

In recent years, the inoculation of bacteria of the genera *Bacillus, Pseudomonas* and *Azospirillum* alone in a hydroponic system has been reported to be beneficial for increasing the root and shoot growth of lettuce, arugula, celery and pac choi plants (Moreira et al., 2022; Oliveira et al., 2023a, b, c, d; Wang et al., 2023; Oliveira et al., 2024; Putra et al., 2024). Prior to these studies, there were no reports of the inoculation of microorganisms in a hydroponic system, either via foliar or nutrient solution, with adequate doses or methods for their inoculation. After these findings, it was possible to evaluate, individually and together, the action of these bacteria inoculated in the nutrient solution in the cultivation of lettuce and arugula hydroponically to demonstrate whether there is a positive synergistic effect of the co-inoculation of two or three bacteria in a hydroponic system.

In our study, it was possible to verify an increase in the length and volume of arugula roots under inoculation with *A. brasilense* and co-inoculation of *A. brasilense + P. fluorescens* compared with those of the non-inoculated plants (**Figures 7A, B**), as well as an increase in root growth of lettuce plants co-inoculated with *B. subtilis + P. fluorescens* compared with those of the non-inoculated plants (**Figure 1A**). The inoculation of *P. fluorescens* and the co-inoculations of *A. brasilense + P. fluorescens* and *B. subtilis + P. fluorescens* increased the volume and fresh mass of lettuce roots compared to the non-inoculated (**Figures 1B, C**). However, co-inoculations with *A. brasilense + P. fluorescens*, *B. subtilis + P. fluorescens* and *A. brasilense + B. subtilis + P. fluorescens* improved root fresh mass of the arugula in relation to that of the non-inoculated plants (**Figure 7C**). Notably, the synergistic effect of the co-inoculation of *A. brasilense, B. subtilis* and *P. fluorescens* on the root growth of lettuce and arugula plants was particularly important. This occurs due to the biostimulant effect of these bacteria, which increases the production of phytohormones such as auxin, which is directly associated with greater root growth (Kang et al., 2019; Zaheer et al., 2022; Kumari et al., 2018). The excretion of some secondary compounds and exudates in the root zone increases root volume and thickness, which increases the capacity for the absorption of water and nutrients by the root system (Verma et al., 2019; Saeed et al., 2021).

Greater root growth, in addition to providing better structuring of plants, is associated with a greater capacity to acquire water and nutrients, which are transported to metabolic routes, tissues, enzymes and cells that are developing and require greater quantities of these minerals (Oliveira et al., 2023a, 2024; Putra et al., 2024). In this way, the increase in the accumulation of N, P, K, Ca and Mg in the lettuce shoot under inoculation with *P. fluorescens*, *A. brasilense + P. fluorescens* and *B. subtilis + P. fluorescens* in comparison to non-inoculated (**Figure 3**). However, the inoculation of *A. brasilense* provided an increase shoot K and S accumulation in arugula plants compared to non-inoculated (**Figure 9B, E**); how, an increase of shoot N and Ca accumulation in arugula under inoculation with *A. brasilense* and *A. brasilense + P. fluorescens* in relation to non-inoculated (**Figure 9A, D**). The inoculation of *A. brasilense + P. fluorescens* and *B. subtilis + P. fluorescens* provided an increase of shoot P and Mg accumulation in arugula plants compared to non-inoculated (**Figure 9C, F**).

This increase in nutrient absorption efficiency may be associated with factors such as increased root growth and ion–root contact, increased photosynthetic capacity, the production of secondary compounds and consequently greater shoot growth and nutrient demand, such as the balanced growth of plants due to the indirect effects caused by plant inoculation (Chu et al., 2020; Oliveira et al., 2023a; Wang et al., 2023). The increase in the absorption and transport efficiency of N, P, K, Ca, Mg and S in lettuce and arugula plants inoculated with *A. brasilense, B. subtilis* and *P. fluorescens* was the main finding in hydroponic systems (Moreira et al., 2022; Gato et al., 2023; Oliveira et al., 2023a, b, c; Putra et al., 2024). However, the positive synergistic effect of the co-inoculation of these bacteria can increase the acquisition of nutrients by lettuce and arugula plants under the effect of co-inoculation (Wang et al., 2023). Furthermore, the effect of inoculation alone can alter N metabolism in plants in hydroponic systems, as reported in other studies (Moreira et al., 2022; Gato et al., 2023; Oliveira et al., 2024).

Because N is necessary for photosynthesis, the synthesis of amino acids, proteins, and phytohormones (cytokinins), which are components of nucleic acids (DNA and RNA) and cell membranes, energy metabolism, and the synthesis of alkaloids, flavonoids, and other secondary compounds that aid in plant defense, the increased production of lettuce plants in response to the inoculation of growth-promoting bacteria may be the cause of the greater accumulation of N in these plants (Xing et al., 2023; Zhang et al., 2023). However, most of the N acquired in a hydroponic system comes from nitric sources due to the toxicity of ammonia when it is supplied in greater quantities, which can become a problem because the main purpose of consuming lettuce and arugula in the natural form and in the form of a salad, in addition to increasing the bitterness of the leaves, excessive consumption by humans is harmful to health (Cocetta et al., 2021; Moreira et al., 2022; Gato et al., 2023; Oliveira et al., 2024).

The conversion of N-NO_3_^-^ and N-NO_2_^-^ into N-NH_4_^+^, proteins, carbohydrates, chlorophyll, amino acids and nucleic acids in plant metabolism is very important because of the harmful effects of N-NO_3_^-^ on human health, especially because arugula and lettuce are frequently consumed fresh around the world (Gato et al., 2023; Oliveira et al., 2024). In our study, there were increases of accumulation of N-NH_4_^+^in the shoot and in the concentration of TAA in the leaves of lettuce plants under inoculation with *P. fluorescens*, and decreases of the accumulation of N-NO_3_^-^ in the lettuce shoot under inoculation with *A. brasilense*, and co-inoculations with *A. brasilense + B. subtilis* and *A. brasilense + B. subtilis + P. fluorescens* compared with those in non-inoculated plants (**Figures 4A, C, F**). All inoculations and co-inoculations resulted in an increase in NR activity in lettuce leaves, with an emphasis on the co-inoculation of *B. subtilis + P. fluorescens* in relation to the non-inoculated (**Figure 4E**). All co-inoculations provided an increase in concentration TC in lettuce and arugula leaves compared to non-inoculated (**Figures 4G**, **10G**). In addition, with an increase in shoot N-NH_4_^+^ accumulation, in NR activity and in TAA concentration in arugula leaves under inoculations with *A. brasilense, P. fluorescens* and *B. subtilis + P. fluorescens* compared to non-inoculated, however, a decrease in N-NO_3_^-^ accumulation in arugula shoot under inoculations with *A. brasilense, P. fluorescens* and *B. subtilis + P. fluorescens* compared to non-inoculated (**Figures 10A, C, E, F**). In this way, it was possible to highlight the positive effect of co-inoculations and inoculations on the activity of NR in the leaves as well as the reduction in N-NO_3_^-^ in other compounds, such as amino acids, carbohydrates and N-NH_4_^+^. This positive effect on plant nitrogen metabolism under bacterial inoculation has been described previously (Schnabel and Sattely, 2021; Kalantari et al., 2022; Oliveira et al., 2024). Inoculation with *A. brasilense, P. fluorescens* and *B. subtilis* increased the affinity for ammonium assimilation and glutamate biosynthesis in the reaction that binds carbon to N metabolism, which is controlled by glutamate dehydrogenase to improve glutamine and glutamate metabolism (Schnabel and Sattely, 2021; Schmidt et al., 2021; Wang et al., 2023).

The reactions of N metabolism increase carbohydrate and amino acid concentrations in leaves, which are related to increased CO_2_ assimilation and photosynthetic pigment contents (Perchlik and Tegeder, 2018; Dormeyer et al., 2019). An increase in the assimilation of N-NO_3_^-^ by plants and its transport to leaf tissues can improve growth, photosynthetic activity and carbon assimilation via photosynthesis for the structural and metabolic functions of nitrogen in plants (Cunha et al., 2024). Interestingly, our results revealed increases in Chl b, Chl T and CAR concentration in the leaves of lettuce inoculated with *A. brasilense, B. subtilis* and *A. brasilense + B. subtilis* in comparison to non-inoculated (**Figures 5B, C, D**). However, there were increases in Chl a, Chl b, and Chl T concentration in the leaves of arugula plants under inoculations with *A. brasilense, A. brasilense + B. subtilis, A. brasilense + P. fluorescens* and *B. subtilis + P. fluorescens* in relation to non-inoculated (**Figures 11A–C**). The increase in the concentration of chlorophylls in leaves is an indication of an increase in the ability of plants to absorb light of different wavelengths and an increase in their photosynthetic capacity (Wang et al., 2022, 2023). This greater photosynthetic efficiency is associated with physiological and metabolic changes resulting from inoculation with plant growth-promoting bacteria. In other studies, it was possible to verify an increase in the concentration of chlorophyll pigments, photosynthetic efficiency, carbon assimilation and accumulation in plant tissues under the inoculation of *A. brasilense, B. subtilis* and *P. fluorescens*, in a hydroponic system and in soil (Cocetta et al., 2021; Hameed et al., 2021; Karpagam et al., 2023; Oliveira et al., 2023b, c; Wang et al., 2023; Oliveira et al., 2024).

The improvement in the water status and photosynthetic capacity of the leaves under the effect of co-inoculation of these bacteria is part of the result of our research, which revealed increases in the *gs* and *Ci* in the lettuce leaves was also observed, as well as an increase in the *gs* and *E* in arugula leaves under the co-inoculation of *A. brasilense + P. fluorescens* compared with the non-inoculated. Furthermore, increases in *Ci* and *A* was observed in arugula leaves under co-inoculation of *A. brasilense + B. subtilis + P. fluorescens* and inoculation *B. subtilis* compared to non-inoculated. However, in lettuce, an increase in the *A* of leaves was observed under inoculations with *A. brasilense, B. subtilis* and co-inoculation with *B. subtilis + P. fluroescens* compared to the non-inoculated. Inoculations of *A. brasilese*, *B. subtilis* and *A. brasilense + B. subtilis* provided an increase in *WUE* in lettuce and arugula leaves in comparison to the non-inoculated (**Figures 6**, **12**). The improvement in the water status of the leaves provided by inoculations and co-inoculations increases the efficiency with which the leaves carry out leaf gas exchange and keep the stomata open for a longer period throughout the day (Karpagam et al., 2023). In this sense, it is possible to highlight that the increase in the efficiency of water use, as well as the improvement in the water status of the leaves provided by growth-promoting bacteria, favored greater active photosynthetic activity in the leaves, as well as greater assimilation of CO_2_ by the plants and its deposition. in the tissues, consequently increasing the mass of the plants (Buckley, 2019; Karpagam et al., 2023; Wang et al., 2023). Furthermore, inoculations favor greater plant growth due to increased water absorption and its use in the transport of minerals, nutrients, carbohydrates, sugars and in the plant’s metabolic processes, increasing efficiency in assimilating and fixing carbon and other minerals in plant tissues and producing plants with greater biomass accumulation (Martínez et al., 2019; Oliveira et al., 2023b; Udeh et al., 2023; Wang et al., 2023; Putra et al., 2024). On the basis of all the results obtained in this research, co-inoculation of *A. brasilense, B. subtilis* and *P. fluorescens* via nutrient mixture has a positive effect on the growth of the shoot and roots and the accumulation of nutrients in the shoot, such as increased photosynthetic activity, carbon assimilation, water use efficiency, stomatal conductivity, chlorophyll concentration, and amino acid and carbohydrate contents, such as increased activity of the nitrate reductase enzyme, which leads to a reduction in nitrate accumulation in the shoots of lettuce and hydroponic arugula.

## 5 Conclusions

The inoculation and co-inoculation of microorganisms in the hydroponic cultivation of lettuce and arugula have proven to be promising strategies for increasing productivity and nutrient use efficiency while reducing the need for synthetic inputs. Despite the extensive literature on the benefits of inoculation with plant growth-promoting bacteria, studies focusing on microorganism co-inoculation in hydroponic systems remain scarce. This study contributes to advancing knowledge in this field by demonstrating that co-inoculation with *Azospirillum brasilense + Pseudomonas fluorescens* and *Bacillus subtilis + P. fluorescens* significantly enhanced plant growth and metabolism, optimizing photosynthesis processes and nutrient absorption.

Furthermore, the results showed that co-inoculation influences nitrogen metabolism, leading to a reduction in nitrate accumulation in the plants-an important factor for food nutritional quality and human health. These findings highlight the importance of biotechnology in sustainable agricultural production, with the potential to reduce the environmental impact of hydroponics and improve the quality of horticultural products.

Given these results, future studies should investigate the application of these strategies in other hydroponically grown vegetables, such as celery, chicory, scallion, and watercress, exploring different microorganism combinations and their effects on plant physiology. Assessing their economic feasibility and efficiency under varying environmental conditions will also be essential for consolidating the adoption of these practices in commercial production.

## Data Availability

The original contributions presented in the study are included in the article/**Supplementary Materials**. Further inquiries can be directed to the corresponding authors.
