# Supplementary material for: Inoculation and co-inoculation of lettuce and arugula hydroponically influence nitrogen metabolism, plant growth, nutrient acquisition and photosynthesis
Source: Front Plant Sci. 2025 Apr 16;16:1547821. doi: 10.3389/fpls.2025.1547821 (PMC12040907; doi:10.3389/fpls.2025.1547821)
Supplement: Supplementary file 1 [file DataSheet1.pdf]

Supplementary Material

1 Supplementary Data

2 Supplementary Figures and Tables

2.1 Supplementary Figures

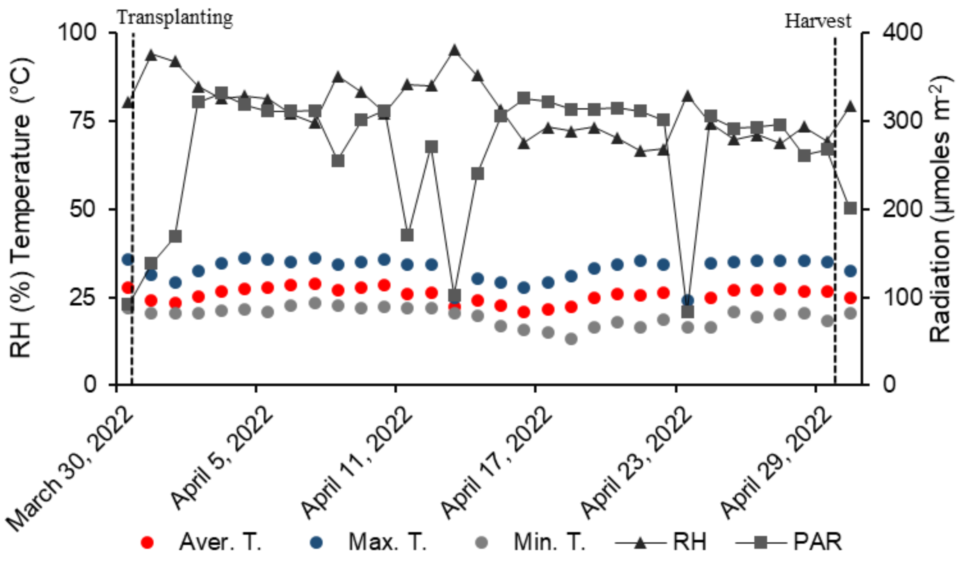

Supplementary Figure 1. The average temperature (Aver. T.), maximum and minimum temperatures (Max. and Min. T.), relative air humidity (RH), and photosynthetically active radiation (PAR) during the experiment.

2.2 Supplementary Tables

Supplementary Table 1. Summary of analysis of variance for probability of root length (RL), root fresh matter (RFM), root dry matter (RDM) and root volume (RV) of lettuce and arugula plants at harvest.

	**Lettuce plants**			
S.V.	RL	RV	RFM	RDM
	cm	mL	------------ g plant^{-1} --------------	
Block	0.69	0.28	0.13	0.71
Inoculation	0.001**	0.000**	0.000**	0.001**
CV (%)	4.93	8.06	3.86	10.32
Mean	31.89	62.16	54.06	3.98
	**Arugula plants**			
S.V.	RL	RV	RFM	RDM
	cm	mL	------------ g plant^{-1} --------------	
Block	0.78	0.51	0.28	0.91
Inoculation	0.001**	0.002**	0.001**	0.001**
CV (%)	7.90	6.15	7.56	8.11
Mean	37.20	31.87	24.95	4.36

** significant at 1%, * significant at 5%, ^{ns} not significant, CV coefficient of variation, S.V. source of variation.

Supplementary Table 2. Summary of analysis of variance for probability of shoot length (SL), shoot fresh matter (SFM), shoot dry matter (SDM) and number of leaves (NL) of lettuce and arugula plants at harvest.

	**Lettuce plants**			
S.V.	SL	NL	SFM	SDM
	cm	N° plant^{-1}	------------ g plant^{-1} --------------	
Block	0.06	0.42	0.15	0.60
Inoculation	0.001**	0.001**	0.000**	0.001**
CV (%)	6.35	10.01	7.61	9.60
Mean	17.50	17.22	349.68	9.89
	**Arugula plants**			
S.V.	SL	NL	SFM	SDM
	cm	N° plant^{-1}	------------ g plant^{-1} --------------	
Block	0.18	0.28	0.59	0.78
Inoculation	0.000**	0.003**	0.002**	0.000**
CV (%)	8.51	11.19	10.88	12.71
Mean	23.99	117.86	110.96	30.95

** significant at 1%, * significant at 5%, ^{ns} not significant, CV coefficient of variation, S.V. source of variation.

Supplementary Table 3. Summary of probability of analysis of variance of shoot nitrogen (N), phosphorus (P), potassium (K), sulfur (S), calcium (Ca), magnesium (Mg) accumulation in lettuce and arugula plants at harvest.

	**Lettuce plants**					
S.V.	N	P	K	S	Ca	Mg
	---------------------------------- g m^{-2} --					
Block	0.09	0.16	0.18	0.08	0.13	0.13
Inoculation	0.001**	0.000**	0.007**	0.010**	0.008**	0.005**
CV (%)	4.06	6.87	8.40	6.91	2.39	20.39
Mean	7.10	0.99	8.74	0.34	1.79	0.85
	**Arugula plants**					
S.V.	N	P	K	S	Ca	Mg
	---------------------------------- g m^{-2} --					
Block	0.21	0.65	0.34	0.22	0.18	0.45
Inoculation	0.002**	0.001**	0.000**	0.003**	0.000**	0.001**
CV (%)	6.28	10.52	9.34	5.27	9.15	10.49
Mean	25.88	4.08	21.93	9.85	8.04	2.00

** significant at 1%, * significant at 5%, ^{ns} not significant, CV coefficient of variation, S.V. source of variation.

Supplementary Table 4. Summary of probability of analysis of variance of shoot ammonium accumulation (S-NH_4^+), nitrate (S-NO_3^-) and root ammonium accumulation (R-NH_4^+), nitrate (R-NO_3^-), leaf NR activity (NR), total amino acids (TAA) and carbohydrates (TC) concentration in lettuce and arugula plants at harvest.

				**Lettuce plants**			
S.V.	S-NH_4^+	S-NO_3^-	R-NH_4^+	R-NO_3^-	NR	TAA	TC
	--------------------------- mg m^{-2} ---------------------------				μmol NO_2^- g^{-1} h^{-1} of FW	------ μmol g^{-1} FW -----	
Block	0.75	0.86	0.65	0.89	0.96	0.98	0.63
Inoculation	0.000**	0.001**	0.000**	0.000**	0.000**	0.000**	0.000**
CV (%)	9.84	4.36	12.58	14.33	6.84	8.21	7.06
Mean	83.34	453.50	37.72	255.77	718.79	9.48	1.70
				**Arugula plants**			
S.V.	S-NH_4^+	S-NO_3^-	R-NH_4^+	R-NO_3^-	NR	TAA	TC
	--------------------------- mg m^{-2} ---------------------------				μmol NO_2^- g^{-1} h^{-1} of FW	------ μmol g^{-1} FW -----	
Block	0.16	0.52	0.49	0.25	0.92	0.48	0.52
Inoculation	0.010**	0.001**	0.001**	0.002**	0.000**	0.003**	0.001**
CV (%)	10.84	10.44	11.25	13.08	5.36	9.35	8.69
Mean	365.91	880.71	126.99	72.05	463.25	11.94	1.96

** significant at 1%, * significant at 5%, ^{ns} not significant, CV coefficient of variation, S.V. source of variation.

Supplementary Table 5. Summary of probability of analysis of variance of shoot ammonium accumulation (S-NH_4^+), nitrate (S-NO_3^-) and root ammonium accumulation (R-NH_4^+), nitrate (R-NO_3^-), leaf NR activity (NR), total amino acids (TAA) and carbohydrates (TC) concentration in lettuce and arugula plants at harvest.

	**Lettuce plants**			
S.V.	Chl a	Chl b	Chl T	CAR
	-------------------------- mg g^{-1} of FW --------------------------			
Block	0.75	0.86	0.65	0.89
Inoculation	0.000**	0.001**	0.000**	0.000**
CV (%)	9.84	4.36	12.58	14.33
Mean	0.28	0.73	1.01	0.15
	**Arugula plants**			
S.V.	Chl a	Chl b	Chl T	CAR
	-------------------------- mg g^{-1} of FW --------------------------			
Block	0.75	0.86	0.65	0.89
Inoculation	0.000**	0.001**	0.000**	0.000**
CV (%)	9.84	4.36	12.58	14.33
Mean	0.28	0.73	1.01	0.15

** significant at 1%, * significant at 5%, ^{ns} not significant, CV coefficient of variation, S.V. source of variation.

Supplementary Table 6. Summary of analysis of variance for probability of intercellular CO_2 concentration (*Ci*), net photosynthesis rate (*A*), stomatal conductance (*gs*), transpiration (*E*) and water use efficiency (*WUE*) in leaves of lettuce and arugula plants at harvest.

	**Lettuce plants**				
S.V.	*Ci*	*A*	*gs*	*E*	*WUE*
Block	0.06	0.42	0.15	0.60	0.87
Inoculation	0.000**	0.001**	0.000**	0.001**	0.001**
CV (%)	7.42	8.01	7.61	9.60	8.45
Mean	386.50	13.58	472.44	7.97	1.78
	**Arugula plants**				
S.V.	*Ci*	*A*	*gs*	*E*	*WUE*
Block	0.25	0.28	0.59	0.78	0.56
Inoculation	0.001**	0.003**	0.002**	0.000**	0.002**
CV (%)	8.19	10.18	10.88	12.71	10.25
Mean	258.05	10.74	313.76	6.75	1.64

** significant at 1%, * significant at 5%, ^{ns} not significant, CV coefficient of variation, S.V. source of variation.
